# Antibody disulfide bond reduction during process development: Insights using a scale-down model process

**DOI:** 10.1186/1753-6561-9-S9-P24

**Published:** 2015-12-14

**Authors:** Julie Ruaudel, Martin Bertschinger, Sonia Letestu, Roberto Giovannini, Paul Wassmann, Pierre Moretti

**Affiliations:** 1 Glenmark Pharmaceuticals, La Chaux-de-Fonds, Switzerland

## Background

Keywords:mAb reduction / disulfide bond scrambling / free thiols

During the development of the production process for a monoclonal antibody (mAb), we observed a significant increase in the reduction of interchain disulfide bonds following pilot scale protein A purification of the produced antibody (IgG1κ).Different companies have reported the presence of fragmented IgG1 antibodies in the clarified cell culture fluid (CCCF) at manufacturing scale. It has been demonstrated [1] that enzymes of the thioredoxin system, released during the decline phase of the culture, were responsible for the interchain disulfide bond reduction. However, our observations were different as the majority of the interchain disulfide bonds were still oxidized in the CCCF. The massive fragmentation of interchain disulfide bonds occurred only after pilot scale protein A purification step. This study presents our insights in antibody reduction using scale-down models.

## Experimental approach

First, the mAb fragmentation phenomenon was reproduced at small scale by incubation of mAb containing CCCF with cell lysate followed by SDS-PAGE with and without NEM (N-ethylmaleimide). NEM is known to protect free thiol groups during sample preparation for SDS-PAGE [2].

Second, a scale down model of the bioreactor process was established in order to understand which process parameters led to the massive reduction and hence fragmentation of the mAb during the pilot scale protein A purification process.

## Results and discussion

**Figure 1:**
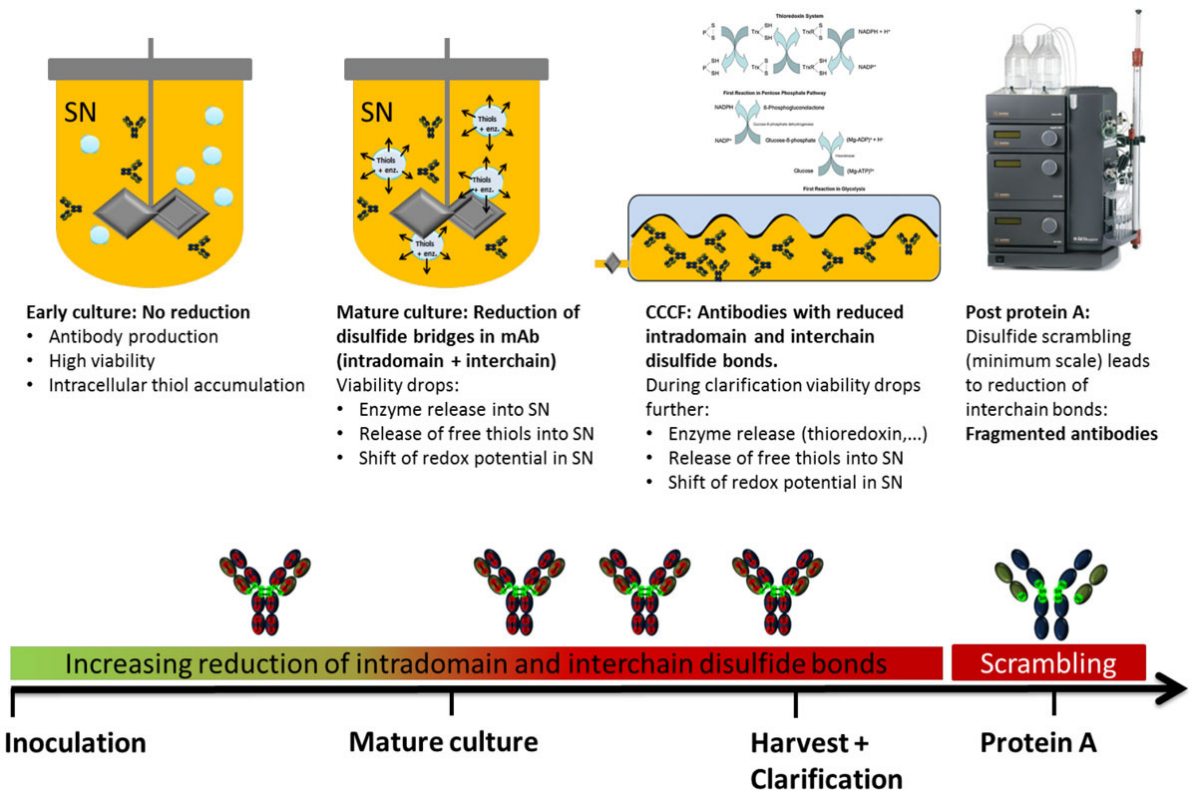
**Our current understanding of the process leading to mAb reduction during pilot scale protein A purification**.

This study demonstrated first that the massive increase in mAb fragmentation could be replicated experimentally in small-scale when the antibody was subjected to protein denaturing conditions. Fully oxidized antibodies were observed in the CCCF using non-reducing SDS PAGE with NEM (protecting the free thiol groups), whereas without NEM, the interchain bonds of the antibodies were massively reduced. We concluded from these experiments that intradomain free thiols can reduce interchain disulfide bonds by disulfide scrambling when these are exposed to denaturing conditions during SDS-PAGE preparation or protein A elution at pilot scale. We also demonstrated that the accumulation of free thiols in the supernatant, observed at the start of the decline phase, led to the increase of free intradomainthiols in the mAb present in the CCCF at the end of the culture. In parallel, the intracellular ratio GSH/GSSG showed a sharp increase from day 7 onwards, indicating a change in the intracellular redox potential in the process. One hypothesis is that these accumulated intracellular reductive forms were released in the supernatant as soon as the viability decreased, leading to a more reductive environment. The detailed mechanisms responsible for this intracellular change remain unclear. Figure 1 summarizes our understandings of the phenomenon.

## Conclusion

MAb reduction is a complex mechanism, which was found in our case to be related to the upstream process rather than a specific cell line. We observed this phenomenon with two different IgG1κ expressing CHO cell lines in two different non-optimized processes, in which the cell viability decreased quickly and sharply. As this viability decline was identified to be the root cause for the release of thiols in the supernatant, the most evident way to prevent the reduction phenomenon might be the modification of the process conditions in order to maintain a good viability throughout the entire upstream process until the clarification step. Further, different IgG formats have been shown to have different sensitivities to reduction [3], thus choosing the right IgG format might be helpful to avoid reduction. The addition of copper sulfate has been also identified as a treatment in the literature [4]. Added at the beginning of the culture, copper sulfate maintains the supernatant in an oxidative state until the end of the culture and thus prevents an increase of free thiols in the supernatant.

## Acknowledgments

All Glenmark teams involved in this project (CLD, USP, DSP, QC, Immunology and AE)
